# Reduced T-cell densities in cranial nerves of patients who died with SARS-CoV-2 infection

**DOI:** 10.1186/s40478-022-01502-9

**Published:** 2023-01-14

**Authors:** Juliane Bremer, Johannes Friemann, Saskia von Stillfried, Peter Boor, Joachim Weis

**Affiliations:** 1 Institute of Neuropathology, Uniklinik RWTH Aachen, Aachen, Germany; 2 Institute of Pathology, Märkische Kliniken GmbH, Klinikum Lüdenscheid, Lüdenscheid, Germany; 3 Institute of Pathology, Uniklinik RWTH Aachen, Aachen, Germany

SARS-CoV-2 patients show a highly variable disease course, ranging from asymptomatic infections to critical illness and death. Most of the fatalities occur in the older patient population and are linked to SARS-CoV-2-induced acute respiratory distress syndrome/diffuse alveolar damage (ARDS/DAD) of the lung, followed by multi-organ failure [20]. In addition to causing ARDS/DAD, SARS-CoV-2 infection may also affect the central and peripheral nervous system. Associations with Guillain-Barré syndrome (GBS), isolated cranial neuropathy, Miller Fisher syndrome and polyneuritis cranialis or chronic inflammatory demyelinating polyneuropathy (CIDP) have been described [3, 4, 6, 18]. Subclinical electrophysiological signs of trigeminal nerve involvement have been demonstrated recently in the majority of SARS-CoV-2 infected patients [2]. In line with clinical findings, in COVID-19 patients with isolated anosmia, the olfactory nerves displayed histological signs of inflammation and damage at autopsy [9] and also the olfactory bulb and tract showed axonal damage in some cases [7]. Although controversial, the olfactory route has been suggested as an entry point for SARS-CoV-2 to invade the CNS [8, 12]. In a subset of patients, viral protein was also detected within other cranial nerves (IX and X) [11]. Based on this, we hypothesized that the cranial nerves of patients who died with SARS-CoV-2 infection might show neuropathological signs of involvement.

We collected formalin-fixed olfactory tract and cranial nerves V, VII and X of 38 SARS-CoV-2-infected patients (24 male, 14 female; 63% male) and 52 control patients (37 male, 15 female; 71% male) at autopsy in the framework of the German COVID-19 autopsy registry [20]. The registry including the retrospective scientific work-up of autopsy-derived biomaterials was approved by the Ethics Committee, Medical Faculty, RWTH Aachen University (EK 092/20 & EK 460/20). There was no significant difference in gender distribution or mean age between the two groups: 66 ± 1.5 years for controls, 69 ± 1.8 years (SEM) in SARS-CoV-2 infected patients. 32 of the 38 SARS-CoV2-infected patients (86%) had contracted the wild-type virus before February 2021, when the alpha variant became predominant in Germany, two patients were infected during the beta wave (February-May/2021), two patients during the delta wave (June-December/2021) and two patients during the omicron wave (end of January and April 2022). At least 31 of the 38 SARS-CoV-2-infected patients (82%) had clinical and/or autoptic signs of ARDS/DAD and/or pneumonia. Clinical signs of cranial nerve involvement including loss of smell had not been reported for any of these patients. However, minor inflammation could remain clinically undetected, particularly since most critically ill COVID-19 patients undergo sedation during invasive ventilation where the clinical status of individual cranial nerves may remain undetected. We focused our analysis on the detection of inflammatory infiltrates as well as signs of neuropathy, this would include, but is not restricted to the detection of inflammatory neuropathies such as GBS [10]. Furthermore, it would allow us to detect peripheral nerve damage or neuroinflammation especially of the trigeminal nerve which has been found to be involved frequently based on electrophysiological data [2]. We performed immunhistochemistry for CD3-positive T-cells along with neurofilament immunohistochemistry and haematoxylin–eosin staining of FFPE tissue of the olfactory tract and cranial nerves V, VII and X (Fig. 1). The average density of CD3+ T-cells within the examined control cranial nerves V, VII and X was 35.7 ± 3.5 T-cells/mm^2^ endoneurial area. T-cell density was highest in the trigeminal nerve (V; 53.2 ± 6.7 (SEM)), followed by the facial (VII; 34.7 ± 5.2 (SEM)) and vagal nerves (X; 21.3 ± 3.0 (SEM)). The olfactory tract, considered to be part of the CNS, showed the lowest T-cell density (13.8 ± 1.8 cells/mm^2^) within the control group. The rather high counts in the trigeminal nerve are in line with the results of a non-quantitative study reporting a “surprisingly” high number of T-cells in the trigeminal nerve and ganglion [13] that might be attributable to persistent herpes simplex virus infection of the trigeminal ganglion [19]. The T-cell counts observed in cranial nerves were similar to those previously reported for sural nerve biopsies (43 ± 13 T-cells/mm^2^) from patients with non-inflammatory neuropathies [1]. Compared to controls, the SARS-CoV-2 patients showed significantly lower densities of CD3+ T-cells in cranial nerves V, VII and X and a trend to lower T-cell densities in the olfactory tract (olfactory tract: 9.6 ± 1.4, V: 34.1 ± 6.9, p = 0.018; VII: 16.9 ± 2.5, p = 0.0009; X: 13.5 ± 3.9, p = 0.0039). We found no correlation between T-cell densities and age and/or gender. Neurofilament immunohistochemistry showed no overt axonal loss. In all nerves with at least 20 T-cells/mm^2^, we also performed CD8-immunohistochemistry, which revealed a high correlation between CD3 and CD8 in both controls and SARS-CoV-2 infected patients. Luxol-fast blue-PAS staining and MBP immunohistochemistry revealed no overt demyelination in any of the nerves with increased T-cell densities. To our knowledge, this data provides the first quantitative analysis of T-cell densities in olfactory tract and cranial nerves V, VII, and X in autopsy tissues. Building on these normative values, we showed that inflammatory infiltration of cranial nerves is not a common finding in SARS-CoV-2 infected patients. Instead, patients dying with SARS-CoV-2 infection show reduced T-cell densities in cranial nerves.

**Fig. 1:**
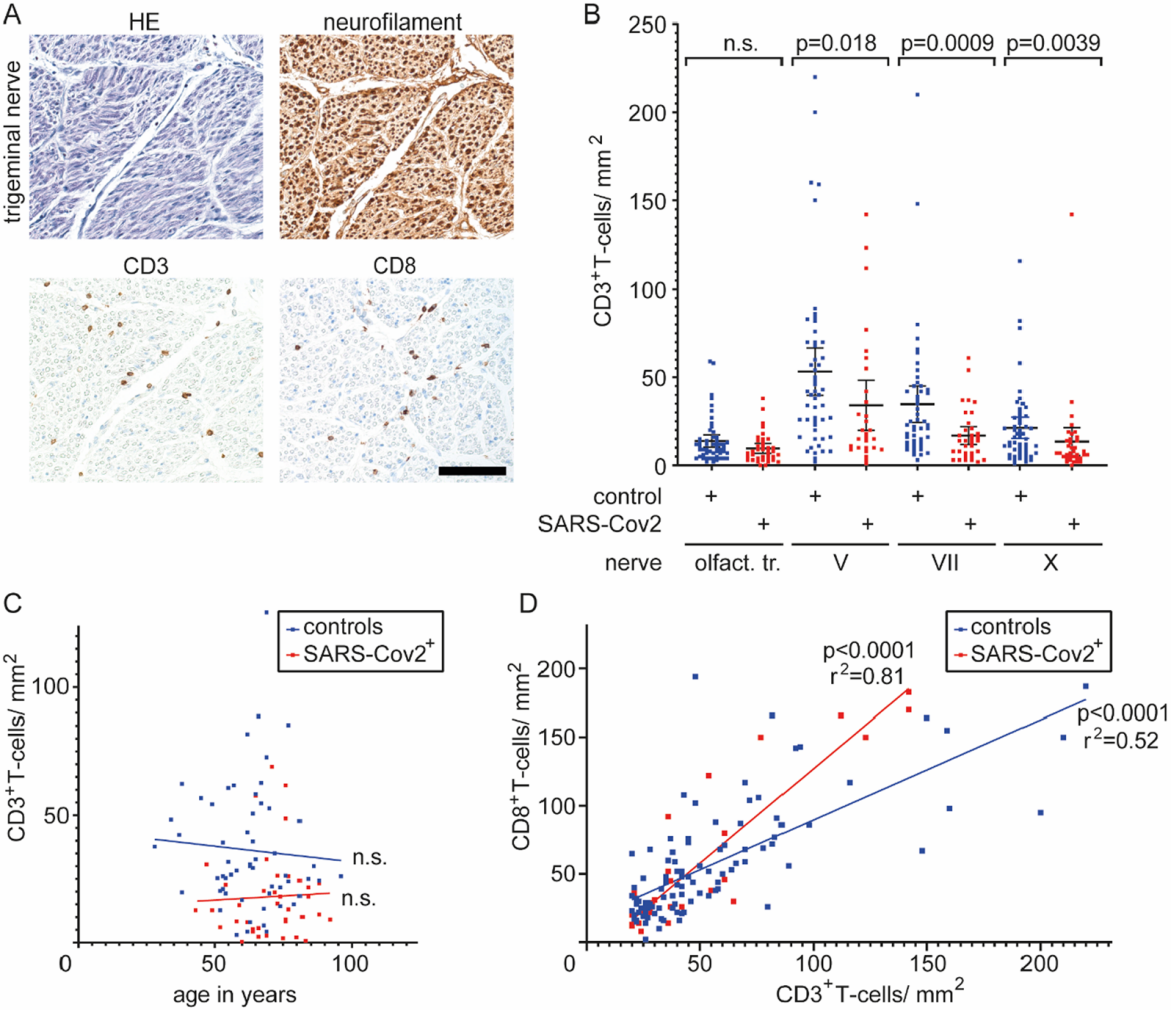
CD3+ T-cells in olfactory tract and cranial nerves V, VII and X in postmortem controls and SARS-CoV-2-infected patients. **a** Conventional haematoxylin–eosin staining (HE) and immunohistochemistry for neurofilament protein, CD3 and CD8 in a cross section of a control trigeminal nerve. Scale bar = 100 µm. **b** Quantification of CD3+ T-cell density in olfactory tract, cranial nerve V, VII and X in controls (n = 52) and SARS-CoV-2 infected patients (n = 38). Mann–Whitney test was used due to unequal variances. **c** No correlation between age and mean CD3+ T-cell density in controls (n = 57) and SARS-CoV-2 infected patients (n = 38); values as mean of CD3+ T-cell density in nerves V, VII and X. **d** Correlation between CD8+ and CD3+ T-cell densities in those nerves V, VII and X with CD3+ T-cell densities of at least 20/mm^2^ in controls and SARS-CoV-2-infected patients

At present we cannot determine the exact pathomechanisms responsible for the reduced T-cell counts, possible explanations include (1) immunocompromised state of patients dying from COVID-19, (2) immunosuppression due to viral infection, intensive care or therapy-related effects and (3) redistribution of immune cells to other organs upon SARS-CoV-2-infection. Peripheral blood lymphocytopenia, especially T-lymphopenia, is seen in many patients with acute SARS-CoV-2 infection, correlates with severe clinical outcomes and is accompanied by the proliferation of the CD8+ T-cell pool, reviewed by [14]. Similarly, peripheral blood lymphopenia is seen in other viral infections [5, 15]. In contrast, lymphocytic infiltration of the lung and other organs has been reported in fatal COVID-19 [16, 17], raising the possibility of T-cell redistribution within the body.

## Data Availability

All numerical data generated or analyzed during this study are included in this published article. Additional information is available from the corresponding author on reasonable request.
